# Comparing Pathways of Bradykinin Formation in Whole Blood From Healthy Volunteers and Patients With Hereditary Angioedema Due to C1 Inhibitor Deficiency

**DOI:** 10.3389/fimmu.2018.02183

**Published:** 2018-10-02

**Authors:** Xavier Charest-Morin, Jacques Hébert, Georges-Étienne Rivard, Arnaud Bonnefoy, Eric Wagner, François Marceau

**Affiliations:** ^1^Axe Microbiologie-Infectiologie et Immunologie, CHU de Québec-Université Laval, Québec, QC, Canada; ^2^Service d'allergie, CHU de Québec-Université Laval, Québec, QC, Canada; ^3^Division of Hematology/Oncology, CHU Sainte-Justine, Montréal, QC, Canada

**Keywords:** B2 receptors, bradykinin, hereditary angioedema with C1 inhibitor deficiency, kallikreins, tissue plasminogen activator

## Abstract

Multiple pathways have been proposed to generate bradykinin (BK)-related peptides from blood. We applied various forms of activation to fresh blood obtained from 10 healthy subjects or 10 patients with hereditary angioedema (HAE-1 or −2 only) to investigate kinin formation. An enzyme immunoassay for BK was applied to extracts of citrated blood incubated at 37°C under gentle agitation for 0–2 h in the presence of activators and/or inhibitory agents. Biologically active kinins in extracts were corroborated by c-Fos accumulation in HEK 293a cells that express either recombinant human B_2_ or B_1_ receptors (B_2_R, B_1_R). Biological evidence of HAE diagnostic and blood cell activation was also obtained. The angiotensin converting enzyme inhibitor enalaprilat, without any effect *per se*, increased immunoreactive BK (iBK) concentration under active stimulation of blood. Tissue kallikrein (KLK-1) and Kontact-APTT, a particulate material that activates the contact system, rapidly (5 min) and intensely (>100 ng/mL) induced similar iBK generation in the blood of control or HAE subjects. Tissue plasminogen activator (tPA) slowly (≥1 h) induced iBK generation in control blood, but more rapidly and intensely so in that of HAE patients. Effects of biotechnological inhibitors indicate that tPA recruits factor XIIa (FXIIa) and plasma kallikrein to generate iBK. KLK-1, independent of the contact system, is the only stimulus leading to an inconsistent B_1_R stimulation. Stimulating neutrophils or platelets did not generate iBK. In the HAE patients observed during remission, iBK formation capability coupled to B_2_R stimulation appears largely intact. However, a selective hypersensitivity to tPA in the blood of HAE patients suggests a role of plasmin-activated FXIIa in the development of attacks. Proposed pathways of kinin formation dependent on blood cell activation were not corroborated.

## Introduction

**Figure 1:**
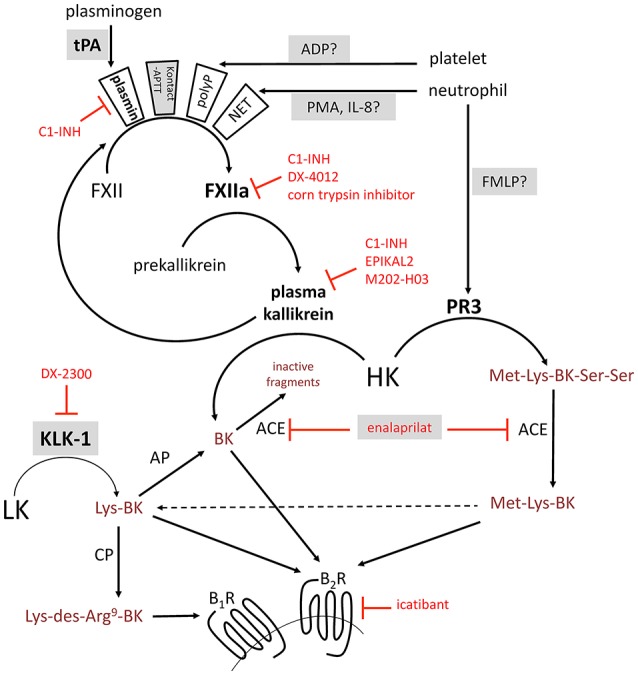
Schematic representation of some of the established or proposed pathways of formation of BK-related peptides. Selected serine proteases are represented in boldface. Selected enzyme inhibitors are represented in red. Stimuli applied in this work to trigger kinin formation in whole blood are named with a shaded background. ACE, angiotensin converting enzyme; AP, aminopeptidase; B_1_R, B_1_ receptor; B_2_R, B_2_ receptor; BK, bradykinin; CP, carboxypeptidase; FMLP, N-formyl-methionyl-leucyl-phenylalanine; FXII, factor XII; HK, high molecular weight kininogen; IL-8, interleukin-8; KLK-1, kallikrein 1 or tissue kallikrein; LK, low molecular weight kininogen; NET, neutrophil extracellular trap; PMA, phorbol 12-myristate 13-acetate; PR3, neutrophil proteinase-3; tPA, tissue plasminogen activator.

The serine protease tissue kallikrein-1 (KLK-1) generates kallidin (Lys-BK) mainly from circulating low-molecular-weight kininogen (LK), whereas plasma kallikrein, a component of the contact activation system, generates bradykinin (BK) exclusively from high-molecular-weight-kininogen (HK) (1, 2) (schematic representation, Figure 1). Both peptides are equipotent agonists of the preformed endothelial B_2_ receptor (B_2_R) (3). Lys-des-Arg^9^-BK, a metabolite produced by carboxypeptidases M and/or N, is the optimal agonist of the inducible human B_1_ receptor (B_1_R) (3). KLK-1 is located in cardiovascular tissue; its release and maturation processes are not fully defined, but this protease is important in abating the consequences of local ischemia, controlling local blood flow (as in flow-dependent vasodilation), and promoting angiogenesis (4).

Decreased expression or function of C1 inhibitor (C1-INH, product of the *SERPING1* gene) is the molecular basis of type 1 and 2 hereditary angioedema (HAE-1, HAE-2), respectively, with ensuing uncontrolled consumption of HK and massive BK generation during attacks (5). Neutrophils are known to be massively activated during HAE attacks (6). The serine proteinase-3 (PR3) secreted by activated neutrophils, reportedly generates Met-Lys-BK-Ser-Ser from HK (7). A synthetic form of this peptide, does not bind to either B_2_Rs or B_1_Rs, but can be converted by peptidases such as angiotensin converting enzyme (ACE) that remove the Ser-Ser C-terminal extension (8), thus generating peptides that can potentially bind B_2_Rs and B_1_R (Figure 1). Further, neutrophils and monocytes reportedly contain KLK-1 that can be secreted (9, 10). Alternatively, the neutrophil extracellular trap (NET) formation by activated neutrophils may also activate the contact system since NETs represent an extracellular surface of histone bound to DNA (11). Other genetic determinants of angioedema states include mutations of factor XII (FXII) and plasminogen genes (*F12, PLG*) (12, 13), presumably leading to fibrinolysis activation (14). The activation of platelets (15) and complement through the lectin pathway (16) may trigger kinin generation as well. Further, drug-induced angioedema/anaphylactoid states, including those associated with ACE inhibitor or recombinant tissue plasminogen activator (tPA) administration, may involve kinin generation and kinin receptor stimulation (17, 18). While ACE is the major kinin-destroying peptidase in the extracellular fluid (19, 20), ACE inhibitor-induced angioedema is reportedly resistant to the plasma kallikrein inhibitor ecallantide and to the B_2_R antagonist icatibant (21, 22).

Drug-induced, hereditary, acquired, or idiopathic angioedema attacks may result from various kinin formation pathways that are perhaps additive, interacting or that have lost inhibitory control. While plasma kallikrein has a central role in HAE-1, HAE-2, and angioedema due to acquired C1-INH deficiency, the role of KLK-1 and cellular elements (leukocytes, platelets) has not been ruled out in the initiation of kinin-mediated vascular disorders. We addressed the formation of BK in whole blood from healthy subjects or HAE patients (HAE-1 or −2 only, sampled during remission) in response to a standardized set of stimuli to validate several of the proposed pathways of kinin formation. An enzyme immunoassay of BK-like peptides was complemented by cell signaling through recombinant B_2_R or B_1_R for confirmation of the generation of active agonist(s). The mechanism of kinin formation was also addressed using modern biotechnological inhibitors of serine proteases.

## Methods

### Human subjects

The local ethical review board (Comité d'éthique de la recherche, CHU de Québec-Université Laval) granted ethical approval to carry out the study involving blood donations from adult healthy volunteers and unrelated HAE patients with C1-INH deficiency (files no. 2016-2263 and 2018-3857). Patient characteristics are listed in Table 1 and their blood donation was obtained during a remission period. Most HAE patients received a prophylactic treatment of plasma-derived C1-INH (Berinert, CLS Behring, Ottawa, Canada), which was not interrupted for the study. Biological evidence of C1-INH deficiency was obtained in the form of antigenic complement C4 levels in patient serum (kinetic nephelometric assay, Dimension Vista system, Siemens, Munich, Germany) and functional C1-INH measurements (based on the inhibitory activity on C1s using a chromogenic substrate, Technochrom C1-INH, Diapharma, West Chester, OH).

**Table 1:** Characteristics of patients with HAE with C1-INH deficiency (3 males, 7 females).

**No**.	**Age range (year)**	**HAE type**	**Approximate frequency of attacks: every**	**Prophylactic treatment**	**C4 (g/l)**	**C1-inh (%)**
1	65–69	1	1 week	Berinert	0.11	30.5
2	25–29	2	6 months	None	0.09	14.1
3	20–24	1	4 months	Berinert	0.12	28.5
4	25–29	1	2 weeks	Berinert	0.15	33.7
5	60–64	1	1 month	None	0.09	12.4
6	55–59	1	4 months	Berinert	0.17	38.5
7	50–54	2	Few days	Berinert	0.20	7.4
8	50–54	2	Few months	Berinert	0.14	46.9
9	15–19	1	Few months	Berinert	0.11	11.4
10	40–44	1	Frequent before prophylaxis	Berinert	0.10	3.9

### Enzyme immunoassay (EIA) of BK

Venous blood was collected without contact with glass in either plastic blood collection tubes with 0.11 M sodium citrate (BD Vacutainer Plus, BD Biosciences, Franklin Lake, NJ) or in blood collection bags containing the same amount of sodium citrate. Fresh blood was kept at room temperature under gentle agitation for 1 h or less. Most BK measurements were performed in whole blood extracts. Each experimental point was obtained using 800 μl of fresh citrated blood transferred to a polypropylene test tube; activators or inhibitors were added to test various pathways of kinin generation (Tables 2, 3); one parallel set of tubes contained the ACE inhibitor enalaprilat (final concentration 130 nM) to protect BK from rapid inactivation (30) since ACE is by far the major kinin-destroying peptidase in the extracellular fluid in plasma and *in vivo* (19, 20). The concentration of KLK-1 used in most experiments (10 nM) is derived from the concentration-response for its contractile effect of the isolated human umbilical vein (31) and also from preliminary experiments addressing iBK release from blood. The usual concentration of Kontact-APTT (20%) is a fraction of that recommended for the coagulation test (50% v/v); that of tPA is taken from Molinaro et al. (18). The tubes were incubated under mild rotary agitation (60–70 rpm) in a 37°C water bath for 0–120 min. At the end of incubation, 4 ml of cold (−20°C) absolute ethanol was added to each tube; this procedure precipitates most proteins, stops kinin generation and catabolism and allows full recovery of kinins in solution (32, 33). The tubes were centrifuged (2,400 g, 20 min, 4°C); the clear supernatant recovered and evaporated overnight to dryness in a SpeedVac apparatus. The extracts were stored at −80°C until used for BK determination; then, they were reconstituted with 800 μl of distilled water, further diluted 100-fold (1,000-fold for selected samples) with the supplied EIA buffer and directly applied in duplicate to the BK EIA as recommended by the manufacturer (Phoenix Pharmaceuticals, Burlingame, CA; cat. no. EK-009-01; 96-well plate format). According to the manufacturer, the antibodies used to detect BK fully cross-react with Lys-BK, but not with des-Arg^9^-BK. We extended this verification to additional sequences (see Results). Whole blood samples stimulated with neutrophil or platelet agonists were submitted to the verification of cellular secretion, NET formation or platelet aggregation (see below).

**Table 2:** Tested stimuli or potentiator of BK formation.

**Stimulus**	**Final concentration in whole blood or plasma**	**Postulated site of activation**	**Source**
Recombinant KLK-1	1–10 nM	LK cleavage	Donated by DiaMedica, Inc.
Pacific Hemostasis Kontact-APTT	4–20% v/v without the calcium supplement	Contact system activation	ThermoFisher Scientific
Cytochalasin B + f-Met-Leu-Phe	5 μg/ml + 1 μM, respectively	Secretion of PR3 from neutrophils (23)	Sigma-Aldrich
Phorbol 12-myristate 13-acetate (PMA)	1 μM	NETosis in neutrophils (11)	Sigma-Aldrich
Recombinant interleukin-8 (IL-8)	25 ng/ml	NETosis in neutrophils (24)	Sigma-Aldrich
Recombinant tPA (alteplase, Cathflow)	169 nM	Fibrinolysis	Roche
Adenosine 5′-diphosphate, sodium salt (ADP)	50–60 μM	Platelet aggregation/secretion (25)	Sigma-Aldrich
Collagen	4 μg/ml	Platelet aggregation/secretion	Chrono-Log
Thrombin receptor-activating peptide (TRAP-6)	10 μM	Platelet aggregation/secretion	Bachem AG
kaolin suspension (CK-Prest Stago reagent)	20% v/v	Contact system activation	Stago
Enalaprilat	130 nM	ACE inhibition	Kemprotec Ltd. (Maltby, UK)

**Table 3:** Tested inhibitors of BK formation/effects.

**Inhibitor**	**Final concentration in whole blood or plasma**	**Postulated site of inhibition**	**Source**
M202-H03: human mAb; close analog of lanadelumab	1 μM	Inhibitor of plasma kallikrein (26)	Donated by Shire Intl. GmbH
EPI-KAL2: recombinant Kunitz inhibitor; close analog of ecallantide	1 μM	Inhibitor of plasma kallikrein (27)	Donated by Shire Intl. GmbH
DX-2300: human mAb	1 μM	Inhibitor of KLK-1 (28)	Donated by Shire Intl. GmbH
DX-4012: human mAb	1 μM	Inhibitor of factor XII (29)	Donated by Shire Intl. GmbH
Corn trypsin inhibitor	4 μM	Inhibitor of factor XIIa	Enzyme Research Laboratories, South Bend, IN, USA
C1-inh (Berinert)	1 U/ml	Inhibitor of plasma kallikrein, factor XIIa	CSL Behring Canada, Ottawa, Canada
icatibant	0.1–1 μM	BK B_2_R antagonist	Donated by Shire Intl. GmbH

### Immunoblots for c-fos signaling

Like other antibody-based assays for BK, the EIA is likely to react with C-terminal BK fragments that have no biological activity (e.g., des-Arg^1^-BK) (32). Therefore, verifying the agonist status of the extracts is necessary. A subclone of HEK 293 cells, called HEK 293a, originally obtained from Sigma-Aldrich (St. Louis, MO) was used in all signaling experiments; these cells do not express significant endogenous populations of either B_1_Rs or B_2_Rs. Cells were grown in Dulbelcco's modified Eagle's medium (DMEM) supplemented with 10% fetal bovine serum, 1% L-glutamine, and penicillin–streptomycin. The agonist action of synthetic peptides and blood extracts on recombinant kinin receptors was investigated in HEK 293a cells using the accumulation of the transcription factor c-Fos, a distal signaling response to the stimulation of various receptor-ligand systems, including the B_2_R (34). Cells stably expressing the human myc-tagged B_2_R (myc-B_2_R) were used (31), whereas the expression vector encoding human B_1_R (construction and validation reported in Figure S1 and its legend, Supplementary material) was transfected in other pre-plated HEK 293a cells as described (35). Cells generally plated 6-well plates (occasionally in 25 cm^2^ flasks) were maintained in 2 ml of culture medium (3 ml for flasks) containing 10% heat-inactivated serum. 48 h after the transfection (for B_1_Rs) or as soon as the cells covered the surface (for myc-B_2_Rs) the adherent cells were stimulated *in situ* for 1 h at 37°C with diluted blood extracts or synthetic kinins. For the analysis of c-Fos in total cell lysates, the culture medium was removed and adherent cells were washed once with ice-cold PBS. Then, boiling lysis buffer containing 10 mM Tris pH 7.4, 1.0 mM Na_3_VO_4_, 1 mM PMSF, one tablet of Complete Mini protease inhibitor cocktail per 10 ml (Roche Diagnostics, Mannheim, Germany) and 1.0% SDS was applied to adherent cells after the removal of PBS. The lysates were removed, transferred to Eppendorf tubes, homogenized by 5 passes through a 27-gauge needle, further incubated for 5 min at 100°C and then centrifuged at 12,000x g for 10 min. Total protein concentrations in supernatants were then determined using the bicinchoninic acid protein assay (Pierce, Rockford, IL, USA). Twenty-five micrograms of total proteins were run on a 9% SDS-polyacrylamide gel electrophoresis and transferred to a polyvinylidene difluoride membrane. The blots were then incubated 1 h at room temperature in blocking buffer [washing buffer (10 mM Tris pH 7.5, 100 mM NaCl, 0.1% Tween 20) containing 5% skimmed milk]. The primary antibody was added for incubation overnight at 4°C in fresh blocking buffer. c-Fos expression was assessed using K-25 rabbit polyclonal antibodies (Santa Cruz Biotechnology; dilution 1:50,000) or a rabbit monoclonal antibody (mAb) (clone 9F6, dilution 1:1,000, Cell Signaling Technology). The membranes were washed for 30 min in washing buffer at room temperature before adding the appropriate secondary antibody (horseradish peroxidase-conjugated, preadsorbed grade; Jackson ImmunoResearch Labs, West Grove, PA) for 1 h at room temperature in blocking buffer. The membranes were washed in washing buffer for another 30 min and then the antibodies were revealed using the Western Blot Chemoluminescence Reagent Plus (NEN Life Science Products), as directed. Equal track loading was further verified by migrating and transferring the same samples separately and immunoblotting for β-actin (mAb from Sigma-Aldrich; dilution 1:50,000).

### ELISA of proteins secreted from phagocytic leukocytes or platelets

To document the secretion of granule contents by neutrophils or platelets in stimulated blood samples, we measured myeloperoxidase (MPO) and platelet factor-4 (PF4), respectively. Tubes containing 800 μl of citrated blood were stimulated for 60 min (MPO) or 5 min (PF4) at 37°C with cytochalasin B + f-Met-Leu-Phe, ADP or IL-8 (same concentrations as in experiments dealing with iBK measurements, see Table 2). Then, the samples were rapidly centrifuged (12,000 g, 1 min), the plasma harvested and stored at −80°C. Thawed plasma samples were diluted 1:100-1:1,000 in the assay buffers and applied to ELISA determination of MPO and PF4 (separate kits from R&D Systems, cat. no. DMYE 00B and DPF40, respectively).

### Aggregometer studies of citrated blood

Whole blood aggregometry and lumiaggregometry were performed using two 2-channel Chrono-Log 700 aggregometers (Havertown, PA). Citrated venous blood (800 μL) was introduced in plastic cuvettes and pre-incubated for 5 min at 37°C with 130 nM enalaprilat, then stimulated in stirring conditions (1,200 rev/min) with 60 μM ADP or 4 μg/mL collagen (Chrono-Log) for 5, 15 or 30 min. Luciferase was added to each sample to monitor ATP release (final total volume: 1 ml) and platelet aggregation was recorded by impedance measurement. The reaction was stopped by adding 4 mL of cold ethanol. Samples were then centrifuged for 15 min at 1,000 g and supernatants were stored at −80°C until analysis. Alternatively, citrated whole blood was preincubated with higher concentrations of enalaprilat (1 or 10 μM) and stimulated with 20% v/v of Kaolin (CK-Prest reagent, Stago, Asnières sur Seine, France), 4 μg/ml collagen, 10 μM of thrombin receptor PAR-1 activating peptide (TRAP-6, Bachem AG, Bubendorf, Germany) or 4 μg/mL collagen + 10 μM TRAP-6.

### Detection of NETs in whole blood stimulated with PMA

One milliliter citrated blood samples from a healthy donor were plated in multiple 35 mm petri dishes to which the DNA stain Sytox green was added (100 nM final concentration, from Molecular Probes-Invitrogen) to detect NETs as cotton-like structures larger than cells and anchored or not to cells (36). PMA (1 μM) was also added in some of the dishes. They were incubated at 37°C for 30 min, and then part of the blood was removed to keep a thin sedimented layer of blood cells. The cells were then observed at 400x magnification and photographed in transmission and epifluorescence using an Olympus BX51 microscope coupled to a CoolSnap HQ digital camera (filters for Sytox green: excitation 460–500 nm, emission 510–560 nm).

### Data analysis

Numerical values are reported as means ± standard deviation (S.D.). Sets of values were compared with ANOVA followed by Dunnett's test when values were compared to a common control group. Pairs of values were compared with Student's *t*-test, or occasionally with Mann-Whitney test if the variances significantly differed (Prism 5.0, GraphPad Software Inc., San Diego, CA).

## Results

### Evidence of biochemical abnormalities in HAE patients

**Figure 2:**
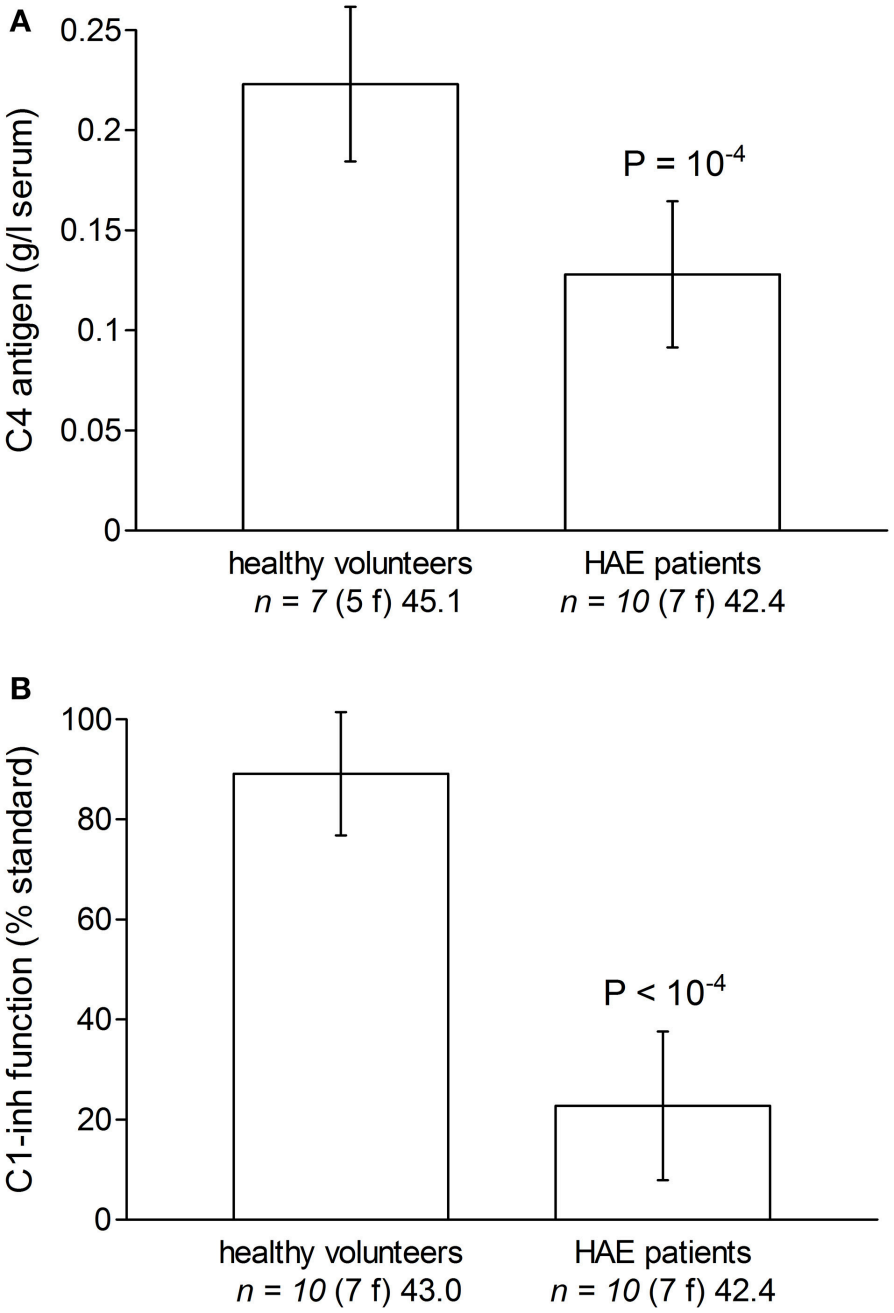
Evidence of biochemical abnormalities in the plasma or serum of HAE patients with C1-INH deficiency included in the study, as compared to these parameters measured in subsets of healthy volunteers. **(A)** Serum C4. **(B)** Plasma C1-inh function (% of normal standard; normal value 70–130% of normal plasma). Values are means ± S.D. Student's *t*-test indicated a significant difference between groups for both C4 and C1-inh (*P*-values reported in Figure). The number of replicates from different donors is indicated by “*n*,” followed by the number of female donors between parentheses (f) and by the average age of donors (years).

Some characteristics of the patients with HAE are displayed in Table 1. While they were seen in the Immunology/Allergy clinic during a remission period and despite the fact that most received Berinert (exogenous C1-INH) prophylaxis, their serum C4 levels and C1-INH functions were significantly lower than those of healthy volunteer blood donors (Figures 2A, B).

### Generation of immunoreactive bradykinin (iBK) in whole citrated blood exposed to various stimulants

**Figure 3:**
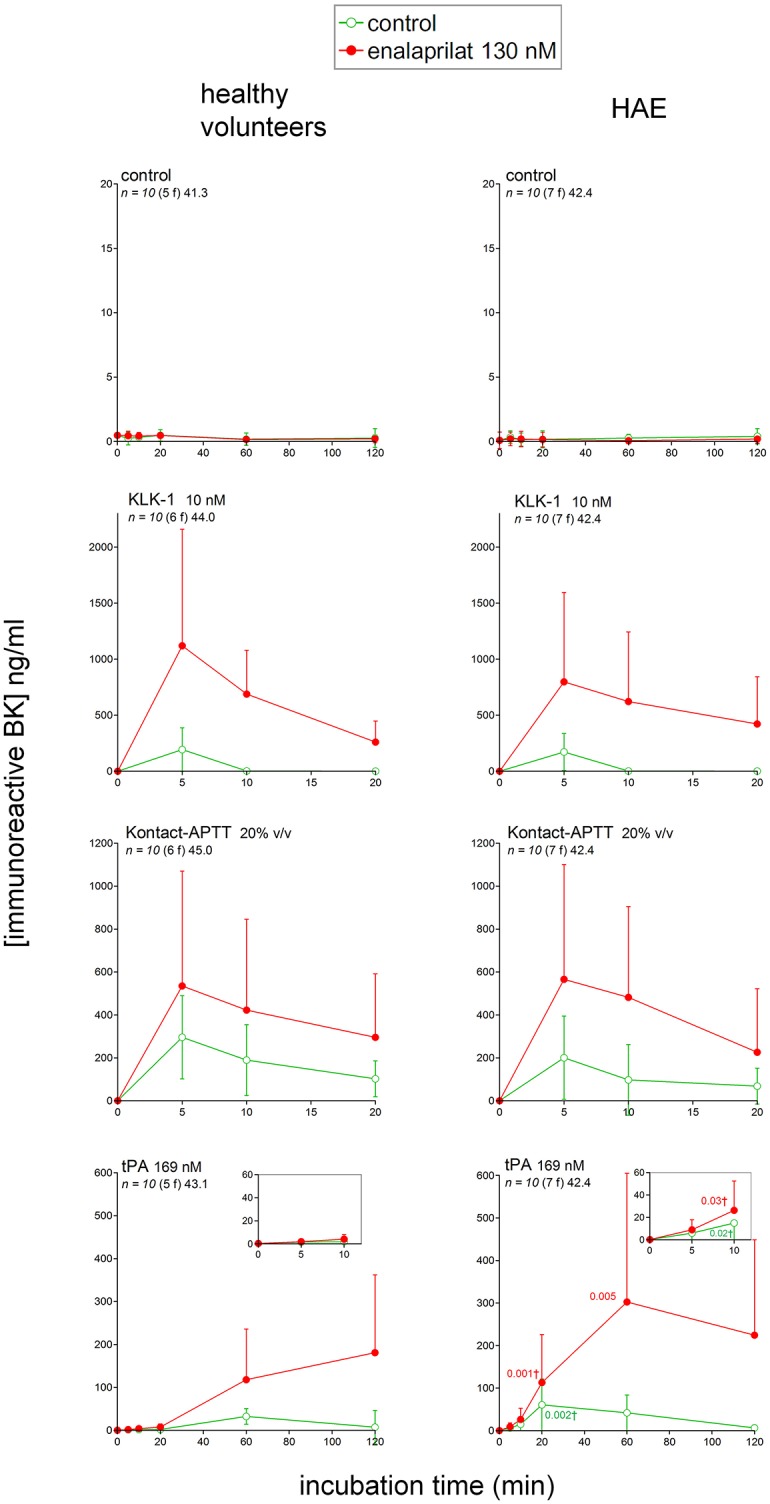
Immunoreactive BK measured in extracts of fresh human citrated blood from healthy subjects or HAE patients with C1-INH deficiency incubated at 37°C under mild agitation in the presence of the indicated stimulus, with optional addition of enalaprilat at130 nM (both applied at time zero). Note the different abscissa and ordinate scales in the various panels, but theses axes are identical on the same lines. Values are means ± S.D. Some errors bars below the average values have been omitted for clarity. The number of replicates from different donors is indicated by “*n*,” followed by the number of female donors between parentheses (f) and by the average age of donors (years). For each experimental condition and time point, Student's *t*-test was applied to isolate the effect of the disease; if variances significantly differed, the Mann-Whitney was applied instead. tPA was the blood stimulus that produced the only statistically different iBK concentrations (significant *P* values for the comparison of normal volunteers with HAE patients reported in the tPA graph applicable to HAE donors; *P*-values followed by †are derived from the Mann-Whitney test).

The concentration of iBK remained low (≤1 ng/ml) in unstimulated blood samples from either healthy volunteers or HAE patients incubated up to 2 h at 37°C, whether or not the ACE inhibitor enalaprilat was added (Figure 3). This applies equally to HAE patients who did not receive Berinert prophylaxis (not shown), despite previous evidence of continuous HK cleavage during remission periods in patients with HAE due to C1-INH deficiency (37). Active recombinant KLK-1 (10 nM) rapidly increased iBK concentration in control blood (≈200 ng/ml, maximal effect for the earliest recorded incubation period of 5 min), but the concentration fell to background level at time 10 min (Figure 3). However, the ACE blocker enalaprilat increased the effect of KLK-1 both in amplitude (≥3-fold at 5 min) and duration (iBK still detectable after 20 min of incubation). Kontact-APTT is a 0.03% magnesium aluminum silica particulate suspension in 1.2% rabbit brain phospholipid solution. It was used here as a contact system activator. The production of iBK in control blood treated with Kontact-APTT (20% v/v) was also very important, but sustained over 20 min, and also further increased by enalaprilat co-treatment (Figure 3). iBK concentrations in extracts of HAE patient blood samples stimulated with either KLK-1 or Kontact-APTT were not significantly different from those of control blood donors (Figure 3).

Fibrinolytic enzymes are suspected to generate kinins in specific clinical circumstances, such as HAE with F12 mutations, in a pathway called contact-independent FXII activation (38). Recombinant tPA (alteplase) very slowly increased iBK in blood samples from healthy volunteers (maximal concentration reached at 60 min), and enalaprilat addition did not hasten the generation, but increased the magnitude and duration of iBK accumulation (Figure 3). The observed kinetics and effect of ACE blockade are consistent with those previously reported in comparable experiments performed with citrated human plasma (same tPA concentration) (18). The downstream fibrinolytic effector, plasmin, may activate the contact system via FXII (39). tPA stimulation in HAE patient blood significantly increased iBK formation (Figure 3); the amplitude of kinin formation was greater than in healthy volunteers, but the slow kinetics was also hasted. The presence of enalaprilat allowed evaluating the formation of iBK with decreased interference from peptide degradation.

**Figure 4:**
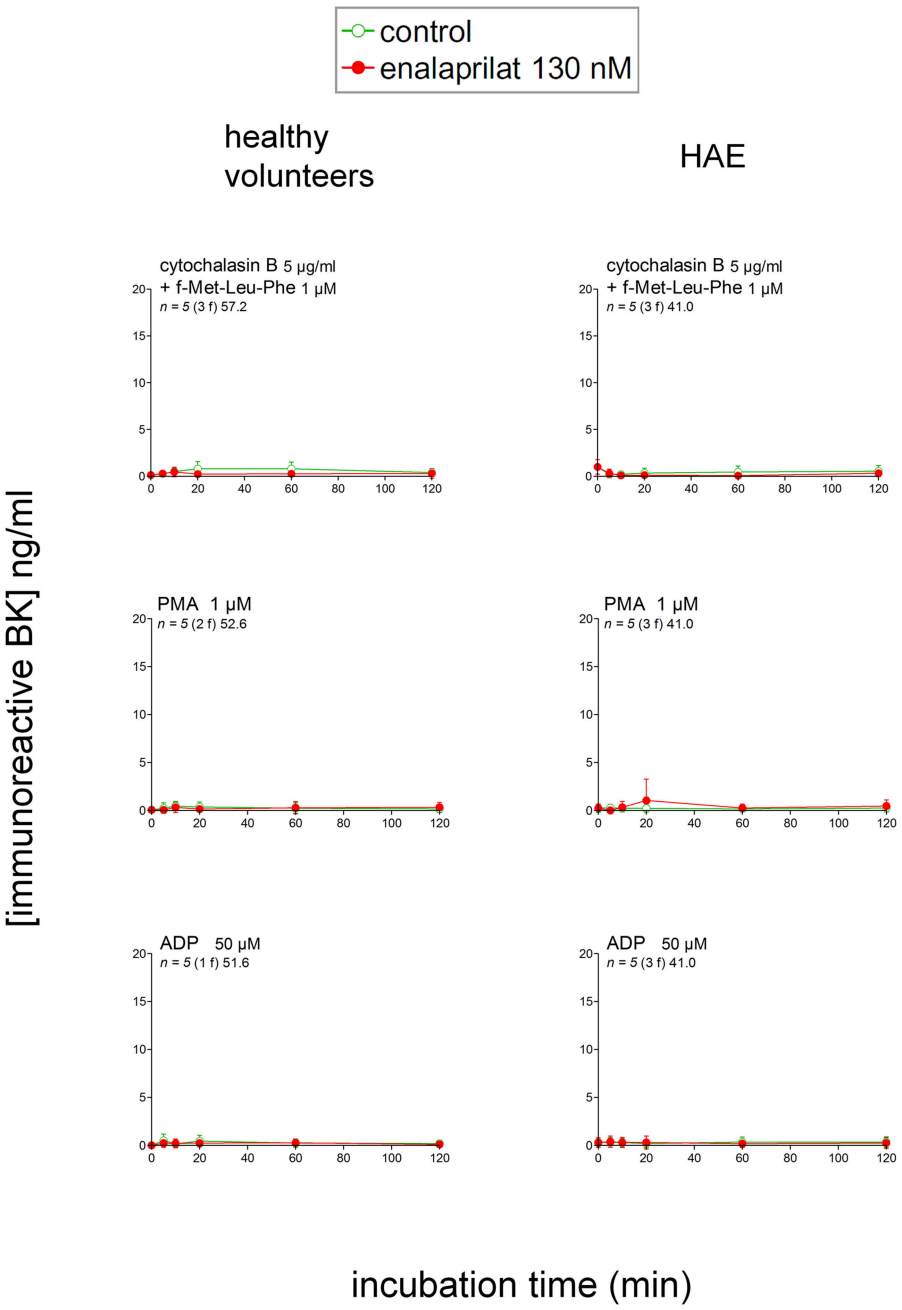
Immunoreactive BK measured in extracts of fresh citrated blood from healthy donors or HAE patients with C1-INH deficiency incubated at 37°C under mild agitation in the presence of the indicated cellular stimulus, with optional addition of enalaprilat 130 nM (both applied at time zero). Values are means ± S.D. and are all similar to the controls reported in Figure 3. Presentation as in Figure 3.

Additional proposed pathways of kinin formation involve specific blood cells; we stimulated phagocytic cells (mainly neutrophils) in fresh blood with a combination optimal for the secretion and/or membrane expression of the serine protease PR3: cytochalasin B + f-Met-Leu-Phe (23). This combination produced no iBK over the control levels in blood from healthy volunteers or HAE patients with C1-INH deficiency (Figure 4). An alternate form of neutrophil stimulation is the relatively slow formation of NETs; f-Met-Leu-Phe is inactive in this respect, but IL-8 reportedly induces NETosis in neutrophils (24) following which the histone-containing DNA NETs may activate the contact system (11). A more vigorous version of NETosis is induced by phorbol 12-myristate 13-acetate (PMA, 1 μM) in human neutrophils (11); however, either PMA or IL-8 stimulation of whole blood from healthy volunteers also failed to produce iBK (Figure 4 and Figure S2). PMA was also inactive on the blood of HAE patients (Figure 4). Interestingly, PMA is reportedly a secretagogue of preformed KLK-1 in neutrophils and monocytes (10). Secretion of anionic polyphosphates from platelets may also activate the contact system under specific experimental conditions (15); a large concentration of ADP (50 μM), theoretically capable of inducing the release of all platelet granule types, failed to increase iBK over baseline in control human blood or that obtained from HAE subjects (Figure 4).

**Figure 5:**
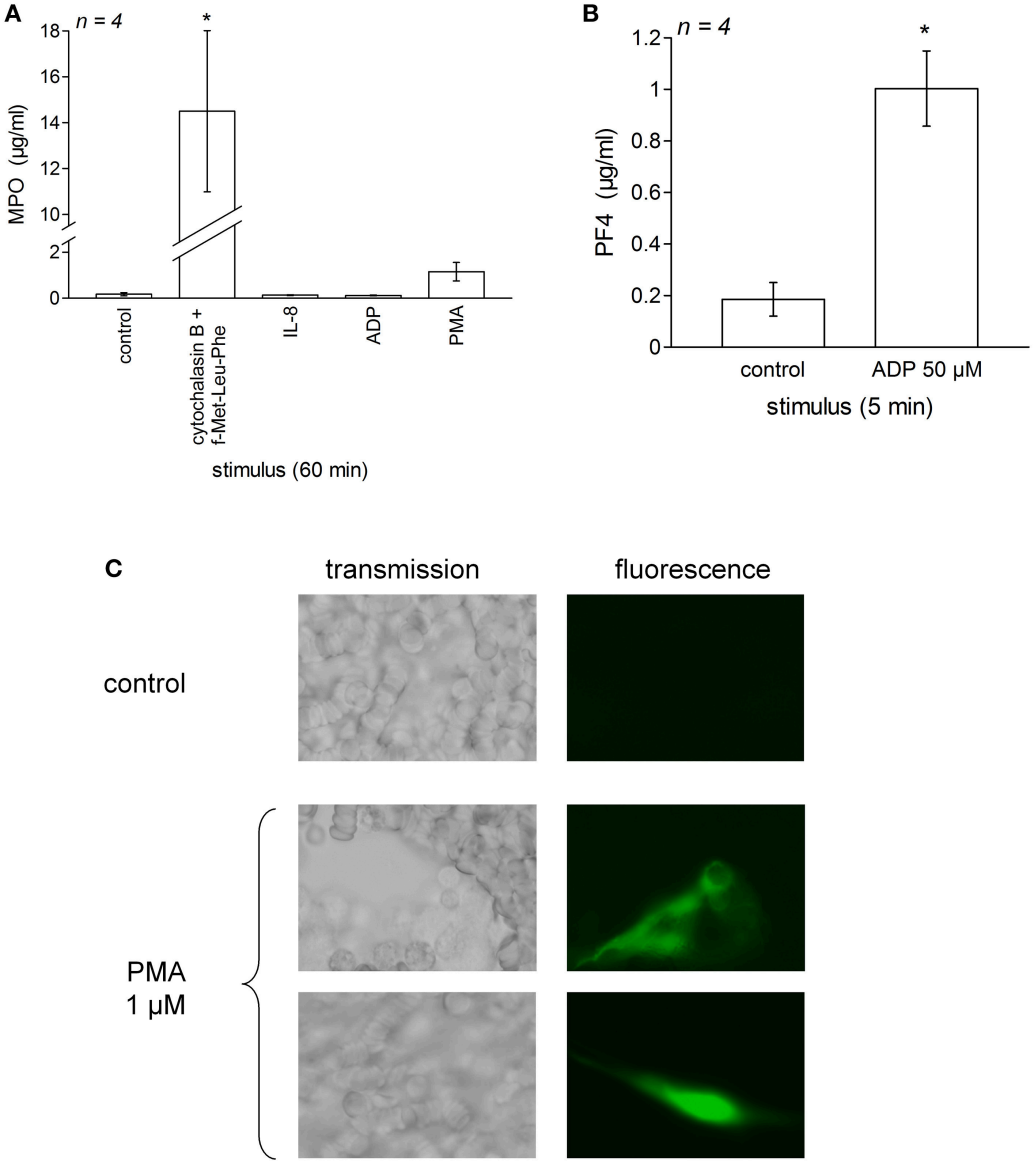
Validation of the secretion of phagocytic cells and platelets in blood samples stimulated as for test of iBK generation. Fresh human citrated blood from healthy donors was incubated at 37°C under mild agitation in the presence of the indicated stimulus (concentrations as in Figure 4 and Figure S2); then the plasma was rapidly obtained and was applied to separate EIAs for myeloperoxidase after 1:100–1:1,000 dilution **(A)** and platelet factor-4 (PF4) after 1:100 dilution **(B)**. Values are means ± S.D. of the number of replicates indicated by “*n*.” In **(A)**, values significantly differed between them (ANOVA *P* < 10^−4^, ^*^P < 0.001 for comparison with the common control value, Dunnett's test). In **(B)**, values also significantly differed between them (^*^P < 0.001, Student's *t*-test). **(C)** Morphological evidence for NET formation from dispersed leukocytes in whole human blood from healthy treated for 30 min with PMA (1 μM, 37°C). Matched fields show transmission and Sytox green epifluorescence (400×).

We verified that typical granule contents were secreted in experiments dealing with stimulants of neutrophils and platelets added to whole blood from healthy subjects (Figure 5). Thus, a very large secretion of MPO into the plasma was recorded in blood samples stimulated for 60 min with cytochalasin B + f-Met-Leu-Phe, but not under IL-8, PMA or ADP stimulation (Figure 5A). ADP stimulated a significant secretion into the plasma of PF4, a plasma marker of platelet activation (40), during a 5-min incubation period (Figure 5B). Morphological evidence of NET formation has been obtained in control blood treated with PMA for 30 min (Sytox green staining of extracellular DNA, Figure 5C). Fluorescent cotton-like structures, anchored or not to isolated cells and much larger than individual cells, were observed only in blood dishes treated with PMA, not in control dishes. While red blood cells are much more numerous than leukocytes and the identity of the cellular source of the NETs is not established in this experiment performed using whole blood, PMA is a highly effective NETosis inducer in neutrophils (11, 24).

**Figure 6:**
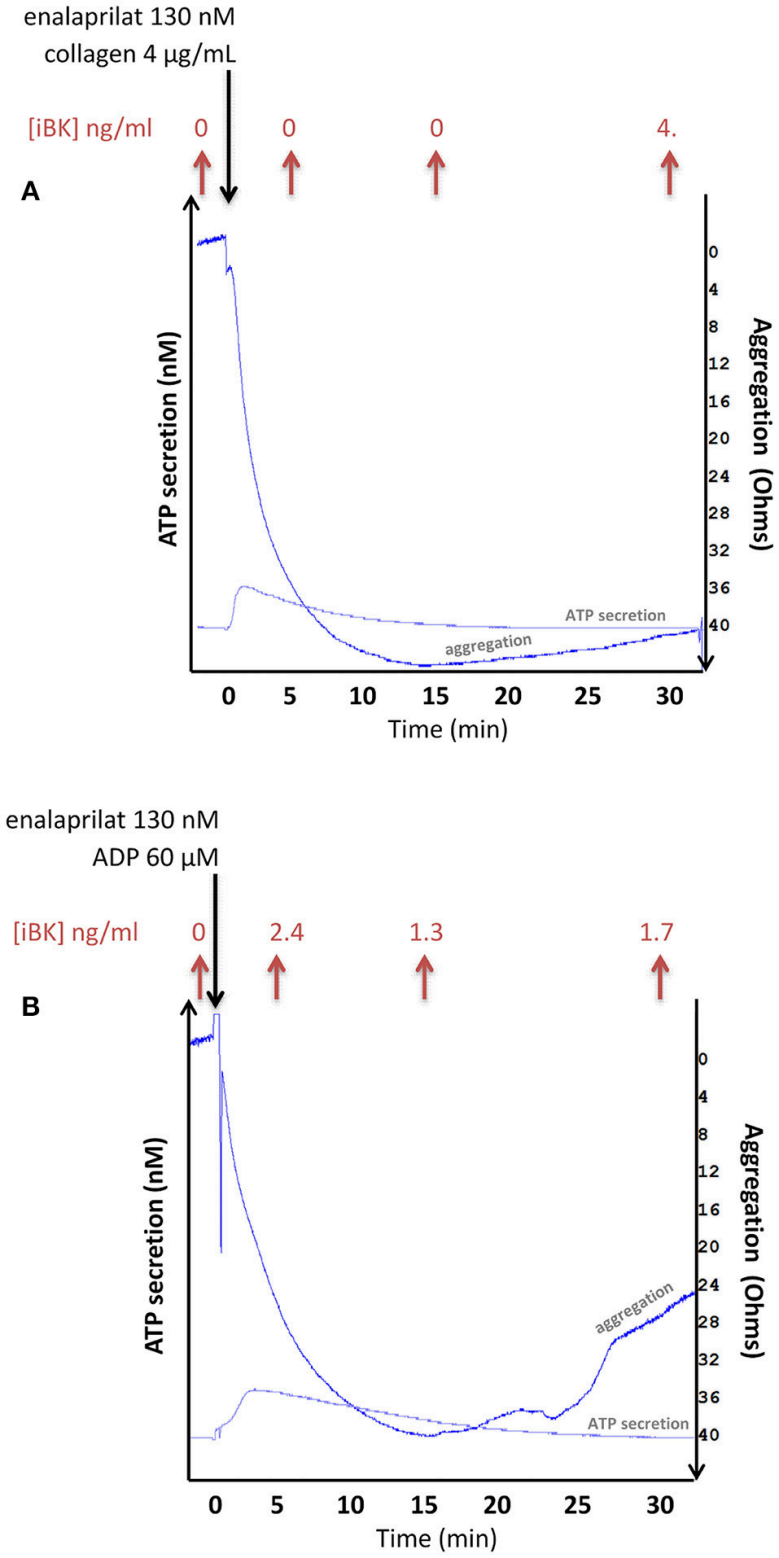
Combined measurement of aggregation, ATP secretion (via luciferase-mediated luminescence) and immunoreactive BK concentration in whole blood samples from healthy donors exposed to stimuli of platelets (**A**: collagen 4 μg/ml; **B**: ADP 60 μM) in an impedance aggremometer. Stirring conditions: 1,200 rev/min. ATP secretion is estimated at approximately 3 nM in both illustrated tracings. The longest recordings are illustrated; others dealing with shorter incubation periods were stopped by the addition of 4 volumes of cold ethanol for the extraction and EIA of iBK. The iBK concentrations, shown on top of each panel, are close to background.

Additional experiments have been performed in whole citrated blood from healthy volunteers maintained in an aggregometer chamber where a shear stress was applied to whole citrated blood samples (Figure 6). ADP (60 μM) or the alternate platelet stimulus collagen rapidly aggregated whole blood *via* platelets as previously suggested (25). A further proof of platelet activation was ATP secretion detected by luciferase-mediated luminescence. Under these controlled conditions, iBK concentrations measured after various incubation periods essentially remained close to background levels (Figure 6), further excluding that platelet stimulation generates kinins in normal blood. Additional iBK measurements performed in blood samples submitted to alternate forms of stimulation in the aggregometer are reported in Table S1. In the presence of very high enalaprilat concentrations (≥1 μM), iBK concentrations remained close to baseline following platelet aggregation with thrombin receptor-activating peptide (TRAP-6), collagen or a combination of both. A kaolin suspension, an alternate activator of the contact system, produced measurable iBK in agitated blood samples (Table S1), but no platelet aggregation.

### Pharmacological validation of biologically active kinins in extracts of blood

**Figure 7:**
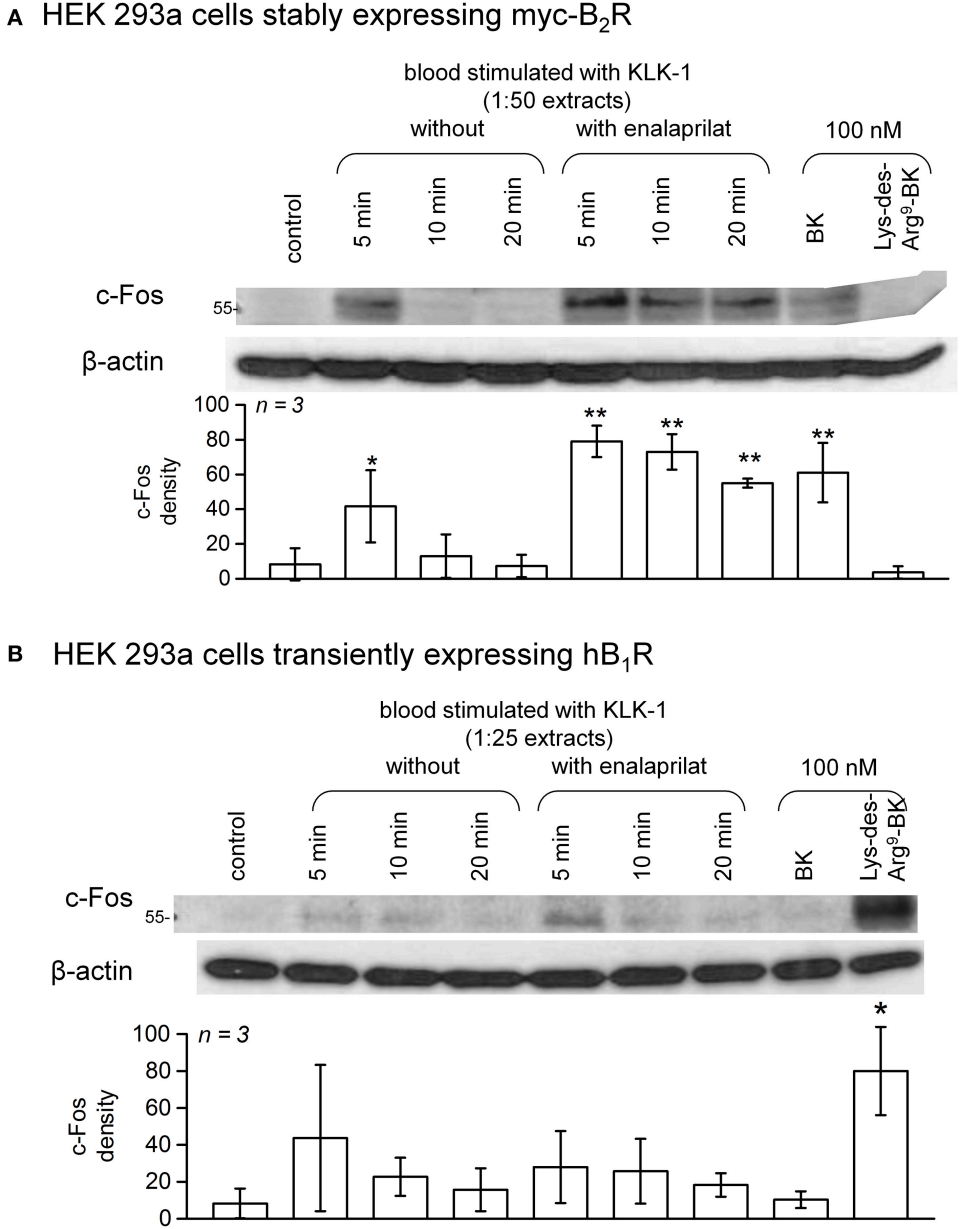
c-Fos accumulation in HEK 293a cells expressing recombinant B_2_Rs **(A)** or B_1_Rs **(B)** and stimulated for 60 min with diluted extracts from healthy subject blood samples incubated in the presence of KLK-1 with or without enalaprilat (blood processed as in Figure 3). Histograms show the c-Fos quantification in replicated experiments (values are means ± S.D., replicate number from separate donors indicated by *n*). In **(A)**, densitometry values significantly differed between them (ANOVA *P* < 10^−4^, ^*^P < 0.05; ^**^P < 0.001 for comparison with the common control value, Dunnett's test). In **(B)**, densitometry values significantly differed between them (ANOVA *P* < 0.01, ^*^P < 0.01 for comparison with the common control value, Dunnett's test).

**Figure 8:**
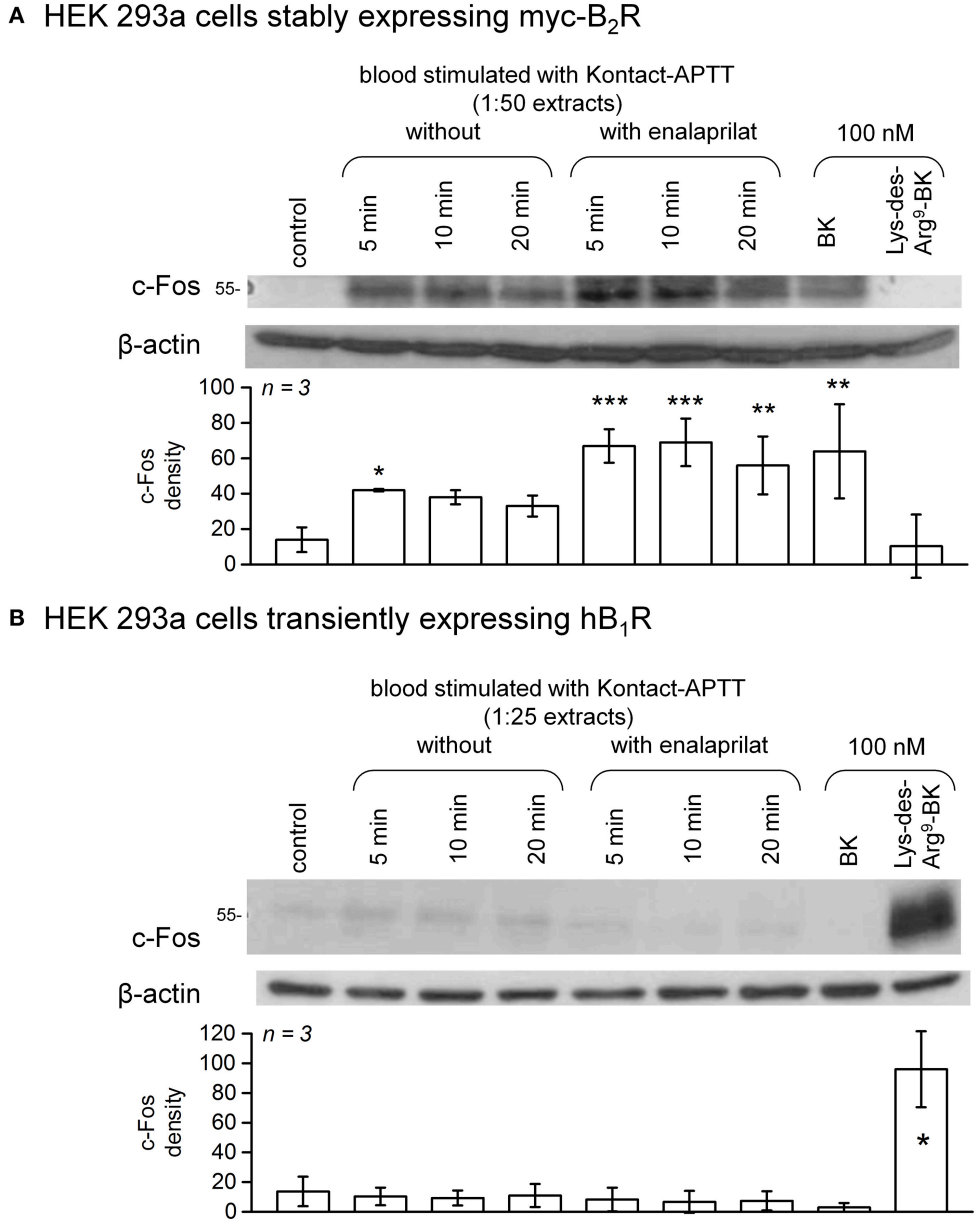
c-Fos accumulation in HEK 293a cells expressing recombinant B_2_Rs **(A)** or B_1_Rs **(B)** and stimulated for 60 min with diluted extracts from healthy subject blood samples incubated in the presence of Kontact-APTT with or without enalaprilat (blood processed as in Figure 3). Presentation as in Figure 7. In **(A)**, densitometry values significantly differed between them (ANOVA *P* < 10^−4^, ^*^P < 0.05; ^**^P < 0.01; ^***^P < 0.001 for comparison with the common control value, Dunnett's test). In **(B)**, densitometry values significantly differed between them (ANOVA *P* < 10^−4^, ^*^P < 0.001 for comparison with the common control value, Dunnett's test).

**Figure 9:**
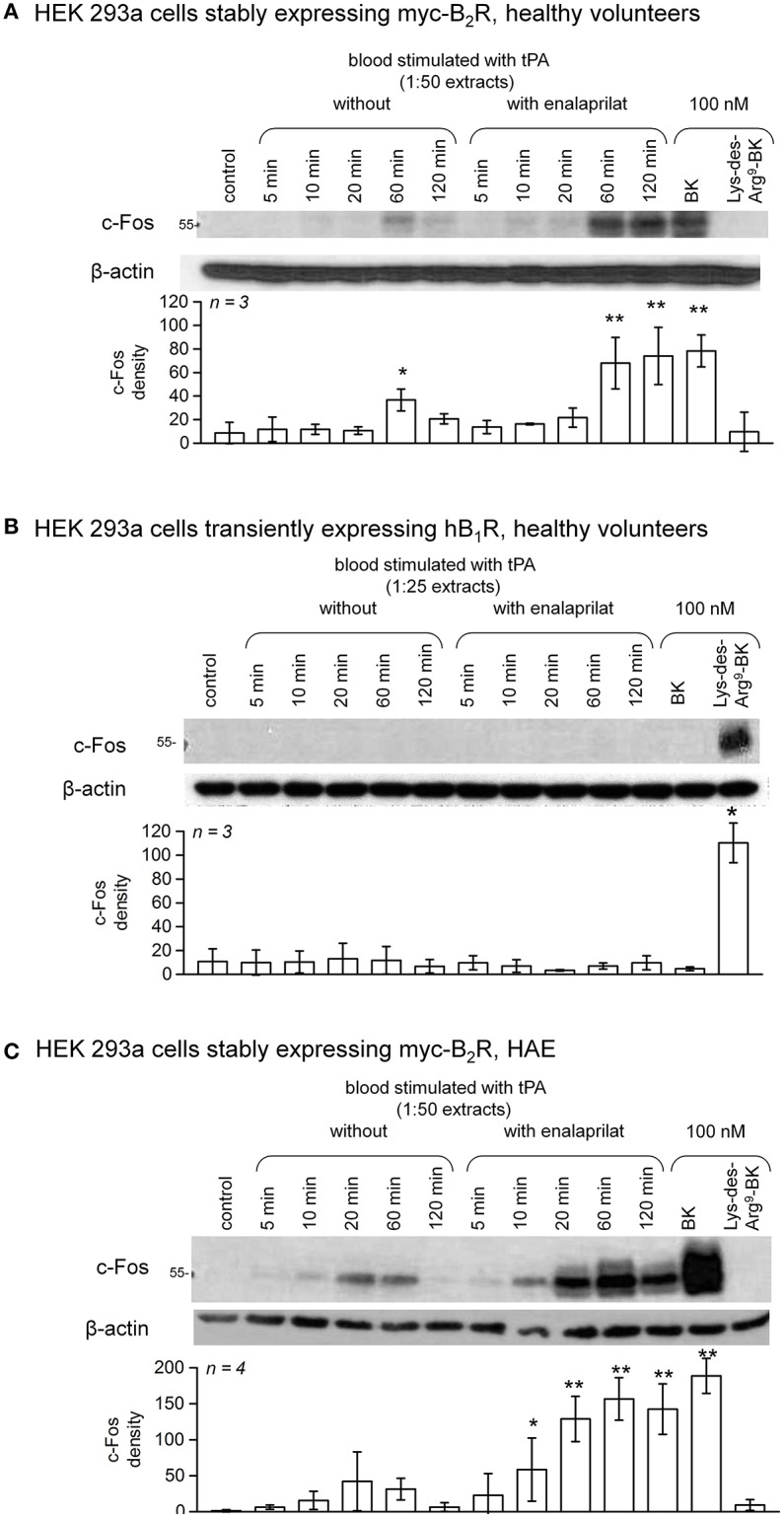
c-Fos accumulation in HEK 293a cells expressing recombinant B_2_Rs **(A)** or B_1_Rs **(B)** and stimulated for 60 min with synthetic peptides or extracts from healthy subject blood samples incubated in the presence of tPA with or without enalaprilat (blood processed as in Figure 3). Presentation as in Figure 7. In **(A)**, densitometry values significantly differed between them (ANOVA *P* < 10^−4^, ^*^P < 0.05; ^**^P < 0.001 for comparison with the common control value, Dunnett's test). In **(B)**, densitometry values significantly differed between them (ANOVA *P* < 10^−4^, ^*^P < 0.001 for comparison with the common control value, Dunnett's test). **(C)** The same assay based on recombinant B_2_Rs was applied to extracts from HAE blood samples incubated in the presence of tPA with or without enalaprilat. Densitometry values significantly differed between them (ANOVA *P* < 10^−4^, ^*^P < 0.05; ^**^P < 0.001 for comparison with the common control value, Dunnett's test).

**Figure 10:**
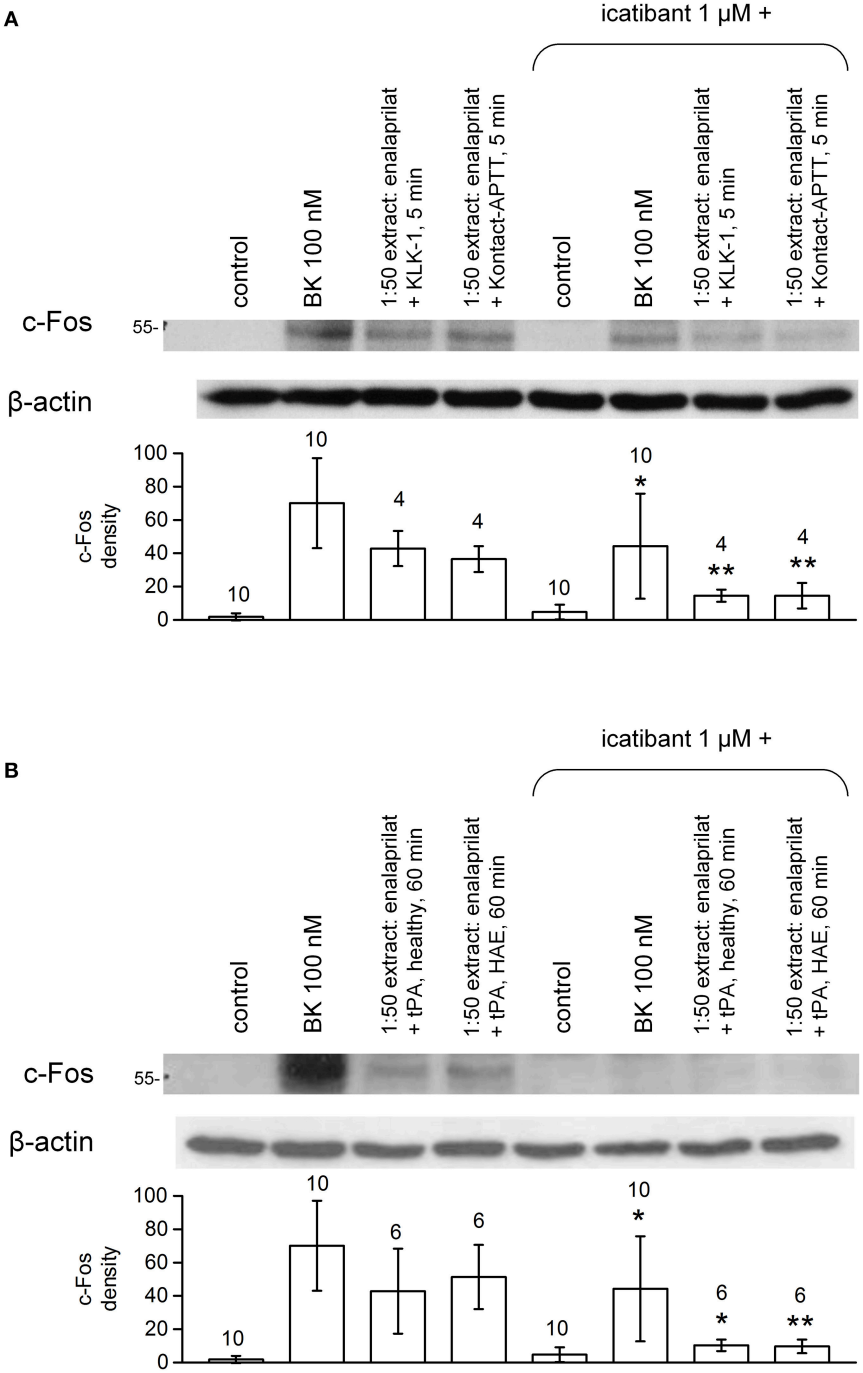
Antagonist effect of the B_2_R antagonist icatibant (1 μM, applied 5 min before extracts) on c-Fos expression induced by extracts of healthy volunteer blood stimulated with enalaprilat along with KLK-1, Kontact-APTT (5 min stimulation, healthy volunteers, **(A)** or tPA (60 min stimulation, both healthy volunteers and HAE patients with C1-INH deficiency, **(B)**. Values are means ± S.D. The effect of icatibant co-treatment is evaluated using Student's paired *t*-test (^*^P < 0.05; ^**^P < 0.01). Numbers above histograms are the number of independent replicates. The controls, performed under the same conditions, have been pooled and the same values are represented in both panels.

According to the manufacturer, the BK EIA exhibits no cross reactivity with des-Arg^9^-BK, but full reaction with Lys-BK, indicating a high discrimination of the C-terminal part of kinins, but little or no selectivity for the N-terminal structure. This reactivity is similar to that of a previously reported antibody-based BK EIA (32). Further, we have established that des-Arg^1^-BK, a fragment devoid of biological activity *via* either B_2_R or B_1_R (41), has full cross-reactivity with the commercial EIA (Figure S3). This indicates a major limitation of the assay: a proof of biological activity was necessary to corroborate the effective presence of an active kinin. We also showed that Lys-des-Arg^9^-BK, the optimal stimulant of the human B_1_R (3), does not cross-react in the EIA (Figure S3).

c-Fos accumulation in cells is a slow and distal response to the stimulation of many receptors, including the BK B_2_R (34). Using HEK 293a cells expressing either human recombinant B_2_R or B_1_R, we investigated the presence of biologically active kinins in whole blood extracts in a semi-quantitative manner (Figures 7–**10** and Figure S4). Firstly, it was confirmed that the B_2_R responds to synthetic BK and Lys-BK (10-100 nM), but not to their respective des-Arg^9^ fragments; des-Arg^1^-BK also failed to stimulate B_2_R signaling (Figure S4A). In the set of synthetic peptides, only Lys-des-Arg^9^-BK convincingly stimulated B_1_R signaling at 10-100 nM (Figure S4B). The assays were applied to diluted extracts from healthy volunteer blood stimulated with KLK-1. As a function of incubation time, they showed a biological activity on recombinant B_2_Rs that paralleled the iBK concentrations (Figure 3) measured in the EIA (Figure 7A). In the absence of enalaprilat, only diluted extracts corresponding to a 5-min incubation period gave a positive signal, whereas the 3 blood samples co-treated with enalaprilat and KLK-1 were positive, but declining from 5 to 20 min of incubation. Extracts from blood samples treated with KLK-1 at a 1:25 dilution induced inconsistent, but occasionally clear B_1_R-mediated c-Fos accumulation, compared to the positive control stimulation using synthetic Lys-des-Arg^9^-BK (Figure 7B). Diluted extracts from Kontact-APTT-stimulated control blood samples (5–20 min incubation) also activated recombinant B_2_Rs, as demonstrated by c-Fos accumulation, with a strong potentiating effect of enalaprilat co-treatment (Figure 8A). However, human B_1_R signaling was not induced by the same extracts in experiments where synthetic Lys-des-Arg^9^-BK was highly active (Figure 8B). The slow effect of tPA on iBK formation in healthy volunteer blood samples (Figure 3) was confirmed by c-Fos accumulation, both in the amplitude and duration when co-treating with enalaprilat (Figure 9A). B_1_R-mediated c-Fos accumulation was not observed in cells treated with extracts of healthy volunteer blood samples treated with tPA (Figure 9B). Extracts from HAE patient blood samples stimulated with tPA were also tested in this assay. Consistent with the EIA results applied to extracts of enalaprilat-treated HAE blood samples, significant B_2_R-mediated c-Fos accumulation was reached for shorter incubation periods vs. extracts of control blood (Figure 9C).

Pretreatment of B_2_R-expressing cells with the B_2_R antagonist icatibant abated c-Fos accumulation following stimulation with extracts from healthy volunteer blood samples incubated for 5 min in the presence of enalaprilat and KLK-1 or Kontact-APTT (Figure 10A) or with extracts from either type of donors stimulated for 60 min with tPA (Figure 10B).

### Differential effects of selected inhibitors of iBK formation pathways

**Figure 11:**
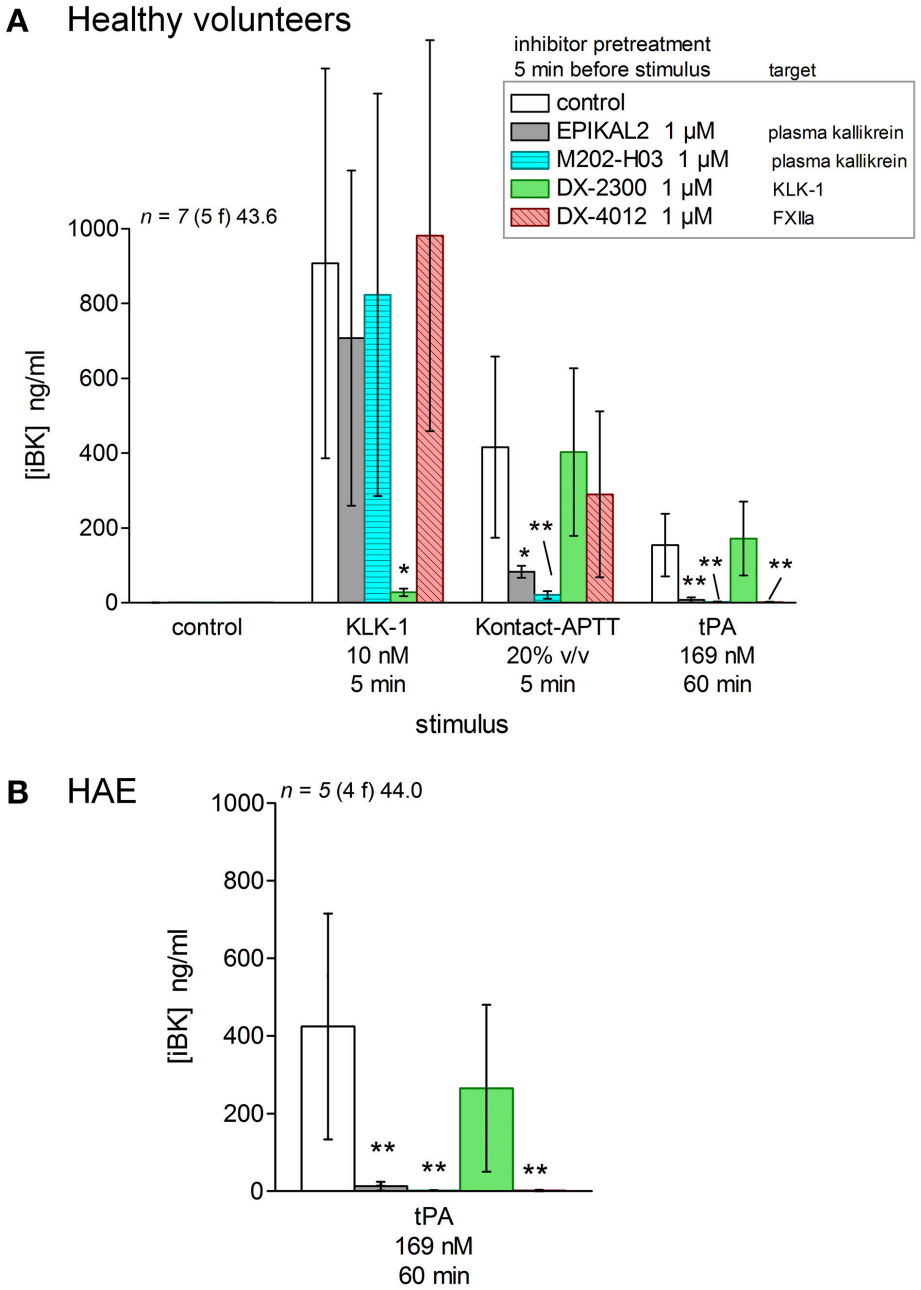
Effect of biotechnological inhibitors, added 5 min before stimuli to citrated blood containing enalaprilat (130 nM), on the generation of iBK induced by KLK-1, Kontact-APTT or tPA (concentrations, incubation periods as indicated). **(A)** Blood from healthy volunteers. Values are means ± S.D. The ANOVA applied to values of iBK from blood under each of the 3 stimuli was significant (*P* < 0.01 for KLK-1, *P* < 0.001 for Kontact-APTT, *P* < 10^−4^ for tPA). ^*^P < 0.01; ^**^P < 0.001 vs. common control (Dunnett's test). **(B)** Blood from HAE patients stimulated with tPA (ANOVA *P* < 0.001; ^**^P < 0.01 by Dunnett's test).

Selected inhibitors were combined with documented activators of iBK formation to dissect out the relationships of stimuli with known or postulated pathways of kinin formations (Figure 1, inhibitors represented in red). Modern biotechnological inhibitors were added to blood samples from healthy volunteers 5 min before one of the 3 active stimuli was added (Figure 11A). KLK-1, Kontact-APTT, and tPA were used at their standardized concentrations (Figure 3) in the presence of enalaprilat (130 nM) and the optimal incubation periods (5 min for KLK-1 and Kontact-APTT, or 60 min for tPA). The KLK-1 active site neutralizing antibody DX-2300 (28) inhibited iBK formation induced only by KLK-1 (Figure 11A). EPIKAL2 is an analog of the peptide ecallantide and M202-H03 is a humanized mAb closely related to lanadelumab (26, 27); both are selective inhibitors of active plasma kallikrein. Accordingly, they both inhibited the effect of the contact system activator Kontact-APTT and of tPA (Figure 11A), indicating that fibrinolysis ultimately recruits the contact system to form BK. These inhibitors did not affect KLK-1-induced formation of iBK. DX-4012 is a humanized mAb directed against the active site of FXII (29); it abated the formation of iBK induced by tPA but, unexpectedly, it was not active against Kontact-APTT. DX-4012 (also termed D06) was shown to inhibit FXII-prekallikrein reciprocal activation in a previous study involving *in vitro* reconstitution and also factor XI-mediated thrombin generation (29). Pending experimental verification, we speculate that DX-4012, a mAb raised against the catalytic domain of recombinant FXIIa, may have a lower affinity for the FXIIa metabolite FXIIf that can activate prekallikrein and kinin formation, but not intrinsic coagulation (1). tPA stimulation produced higher iBK concentrations in the blood from HAE patients than in blood from healthy volunteers, but the same pattern of inhibition was observed in the presence of biotechnological proteins (Figure 11B).

**Figure 12:**
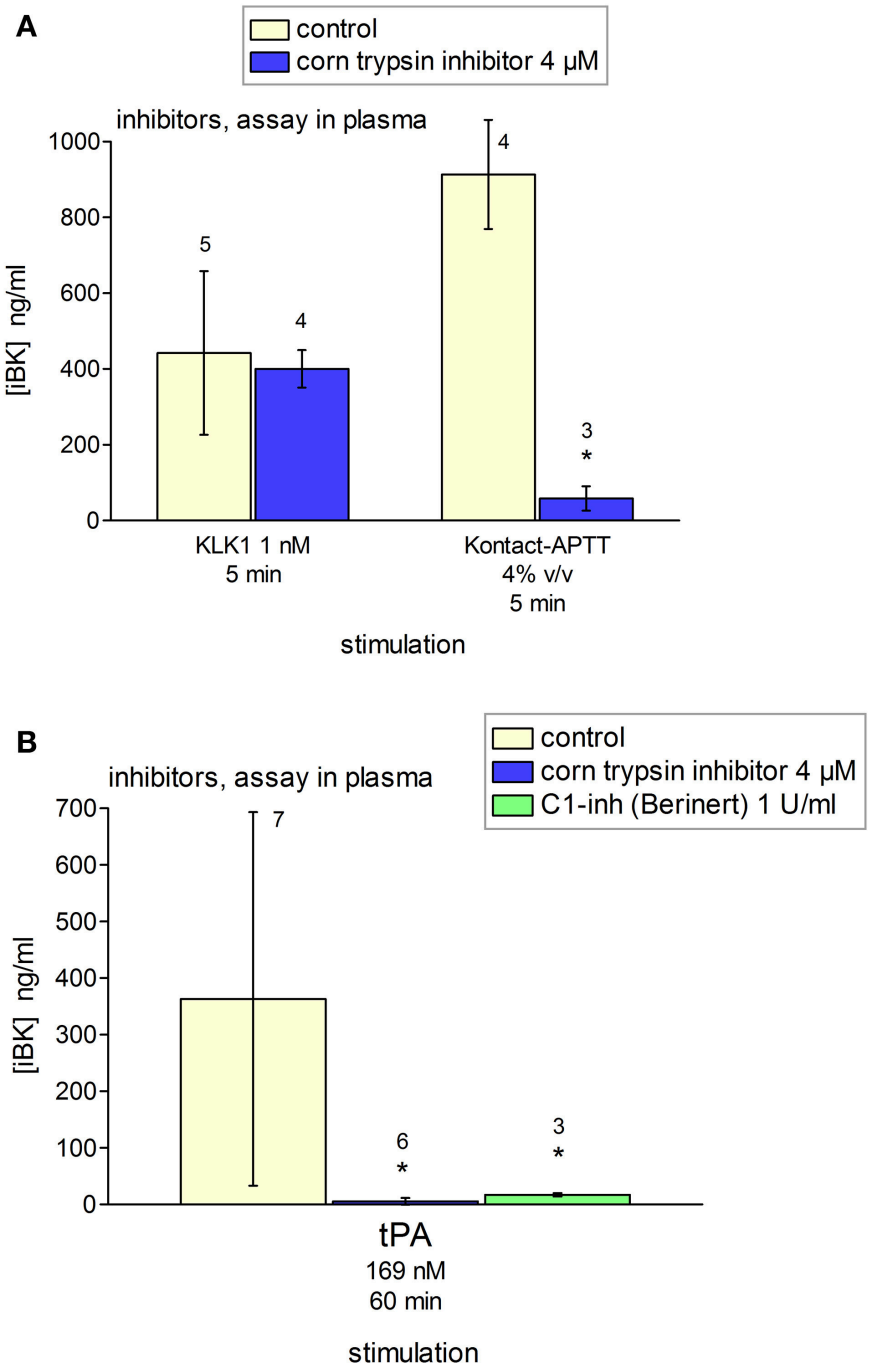
Effect of selected inhibitors added to citrated plasma from healthy donors containing enalaprilat (130 nM) on the generation of iBK induced by KLK-1, Kontact-APTT or tPA (concentrations, incubation periods as indicated, and replicate numbers found above histograms). **(A)** Corn trypsin inhibitor did not inhibit KLK-1-induced generation of iBK; however, it reduced significantly that induced by Kontact-APTT (*t*-test, ^*^P < 0.001). **(B)** Values are means ± S.D. ANOVA indicated significant differences between groups in the values related to tPA stimulation (ANOVA *P* < 0.05; ^*^P < 0.05 vs. common control by Dunnett's test).

Additional inhibitors were tested using blood plasma from healthy volunteers containing enalaprilat (130 nM) (Figure 12). Corn trypsin inhibitor suppresses the enzymatic activity of FXIIa (42), but also that of tPA at similar concentrations (43). This inhibitor significantly reduced tPA- and Kontact-APTT-induced iBK formation (Figures 12A,B). C1-INH (1 U/ml) also significantly abated the production of iBK induced by tPA at 60 min, further supporting the mediation of the contact system. The effect of KLK-1 was not inhibited by corn trypsin inhibitor (Figure 12A).

## Discussion

The scope of the present study is to investigate the formation of biologically active kinins in human blood. Few of the proposed kinin generation pathways have been corroborated in whole blood from healthy subjects or patients with HAE-1 or−2. Kontact-APTT was one of the robust stimuli of iBK formation (Figure 3). HAE-1/2 attacks clearly involve the contact system and are relieved by ecallantide “on demand” treatments (44). Lanadelumab prophylaxis was also effective in a recent phase 1b trial (45). We used closely related biotechnological inhibitors of plasma kallikrein in our *in vitro* analyses, EPICAL2 and M202-H03, respectively. A B_2_R agonist, presumably BK, is present in extracts of Kontact-APTT-stimulated blood from healthy subjects (Figure 8A), but signaling *via* human B_1_R was not observed; the metabolite des-Arg^9^-BK has only a low affinity for the human form of this receptor (3). While HK is reportedly cleaved continuously in HAE patients during remission (37), no baseline iBK formation was evidenced in their blood, whether or not ACE was blocked (Figure 3), possibly indicating the absence of low intensity physiological trigger(s) *in vitro*. Further, iBK formation under Kontact-APTT or KLK-1 stimulation was essentially similar in the blood of HAE patients or healthy subjects. The ecallantide and lanadelumab surrogates, EPICAL2 and M202-H03, were potent inhibitors of iBK formation induced by Kontact-APTT (Figure 11). Icatibant abated the pharmacological effect of the corresponding extracts on B_2_R (Figure 10), in line with proven HAE therapies.

tPA slowly generated iBK and the corresponding blood extracts were biologically active on recombinant B_2_Rs in our experimental system where no surface that can activate the contact system was provided (Figures 3, 9). A form of drug-induced angioedema/anaphylactoid reaction that is occasionally produced by fibrinolytic agents such as tPA may be dependent on contact system components, as shown by the inhibition of tPA-induced iBK formation by M202-H03, DX-4012, corn trypsin inhibitor and C1-INH (Figures 11, 12). One key finding of the present study is the much greater and earlier formation of iBK in tPA-stimulated blood of patients with HAE (Figures 3, 9C). The present data suggest that C1-INH deficiency does not make the contact system unstable, but rather makes it hypersensitive to plasmin. The place of fibrinolysis as a trigger in kinin-mediated angioedema states remains to be investigated in detail; it has been noted that plasmin can activate FXII in the absence of a contact system-surface (38, 46). Tranexamic acid was predictably effective in a subtype of HAE due to a mutation in FXII, although the series is small (47). The identified trigger factors of attacks in HAE with C1-INH deficiency include physical exertion, mechanical trauma, infection, menstruation, dental procedures, etc. (48). This may suggest that inflammation has a triggering role. However, the second more frequent trigger is mental stress (48). Mental stress activates in a predictable manner tPA secretion and the turnover of fibrin (detected by D-dimer formation) (49), indicating that fibrinolysis may be a better conductive thread than inflammation when triggers of angioedema attacks are considered. Mechanistically, tPA is released from endothelial cells by catecholamines, shear stress and BK itself (50–52), in the latter case a potential pathologic feedback in HAE. Remarkably, there is no increased risk of thrombosis associated with HAE with C1-INH deficiency, despite the central role of FXIIa (38). This may be explained if fibrinolysis precedes and determines attacks.

KLK-1 is not usually mentioned as a mediator of angioedema states, despite its very effective action as a kinin-forming agent that is unaffected by HAE-1/2 (Figures 3, 7). Studies with inhibitors indicate that KLK-1 is part of a completely distinct kinin formation system itself not recruited by or not recruiting the contact system (Figures 11, 12). Leukocytes have been proposed to contain preformed KLK-1 (9, 10), and there is evidence for *in vivo* neutrophil activation during HAE attacks (6), suggesting a possible amplification system for kinin generation. However, the massive leukocyte stimulation with FMLP or PMA in the present experiments was not parallel to iBK formation. ACE inhibition generally increased iBK formation without inducing it *per se* (Figure 3). KLK-1 release from vascular or other cell types could be a trigger for ACE inhibitor-induced angioedema, a condition reportedly resistant to ecallantide and probably to the B_2_R antagonist icatibant (21, 22). Ecallantide is a highly selective inhibitor of plasma kallikrein (53). If KLK-1 is the trigger for this form of drug reaction, a contribution of the B_1_R to the vascular physiopathology is possible, as we showed that the activation of the human B_1_R is restricted to KLK-1 stimulation (Figures 7–9). Speculatively, KLK-1 could also be a trigger for yet to be discovered forms of shock or angioedema with normal C1-INH. Thus, the consumption of the KLK-1 inhibitor kallistatin during experimental septic shock has been reported (54).

Pathways of kinin formation involving phagocytic leukocyte and platelet activation were not corroborated when tested in blood from healthy volunteers or HAE patients. While previously proposed molecular triggers of kinin formation, PR3 activity and polyphosphate exocytosis, has not been measured in whole blood experiments, evidence of platelet and neutrophil activation, including granule secretion and NETosis, has been obtained. The applied cell stimuli have wide effects, such as elastase and cathepsin G secretion from FMLP-stimulated neutrophils (23) and KLK-1 secretion from PMA-stimulated neutrophils and monocytes (10) in experiments dealing with purified cell populations. A number of factors may explain the discrepancies, for instance the original observation having been performed *via* reactions with purified proteins (7), or other *in vitro* procedures that exclude the multiple endogenous inhibitors present in whole blood. Neutrophil PR3 is inhibited by α1-antitrypsin (serpin A1) (55), KLK-1 by kallistatin (serpin A4) (56), and the contact system in general by C1-INH (serpin G1) (5). Thus, the genetic or acquired deficiency in one of these or other inhibitors, such as plasminogen activator inhibitor 2 (serpin B2) (57), may incriminate a specific pathway in some patients. A possible limitation of the present study is the lack of a suitable surface for the assembly of a kinin-forming complex. Kontact-APTT, being a particulate material, does not suffer from this limitation, but it may apply to some secretory products of cells, such as polyphosphates.

Despite the prophylactic treatment applied to most patients, the selective hypersensitivity to tPA in the blood of HAE patients is reproducible, suggesting a role of fibrinolysis and surface-independent FXIIa in the development of attacks.

## Author's note

Presented in part at the 10th C1-INH Deficiency Workshop, Budapest, Hungary, 18-21 May 2017.

## Author contributions

XC-M executed most experiments and performed preliminary analyses. JH selected patients. G-ÉR and AB planned and executed most experiments dealing with platelet stimulation. EW worked on the biological characterization of patients. FM, G-ÉR, JH, and EW designed the experiments. FM analyzed results and wrote the manuscript.

### Conflict of interest statement

JH served as speaker/teacher for CLS Behring, Novartis, Shire and Aralez; served on board of advisory committees for AstraZeneca, CLS Behring, Shire, and Novartis; served as clinical investigator for Circassia, Merck (ALK), Stallergene, Boehringer-Ingelheim, GlaxoSmithKline (GSK), Teva, Novartis, Sanofi, AstraZeneca, Johnson & Johnson, CLS Behring, Shire, Roche, Green Cross, Griffols, outside the submitted work. G-ER served on advisory boards for Baxalta, Bayer, Biogen Idec, CSL Berhing, Novo Nordisk, Octapharma, Pfizer and received research funds from Bayer, CSL Behring and Pfizer, outside the submitted work. AB has served on advisory boards for CSL Behring, Novo Nordisk and Alexion Pharmaceuticals, outside the submitted work. He has also received honoraria or research funds from Stago, Alexion Pharmaceuticals and Novo Nordisk outside the submitted work. The remaining authors declare that the research was conducted in the absence of any commercial or financial relationships that could be construed as a potential conflict of interest.
