# Supplementary material for: Comparing Pathways of Bradykinin Formation in Whole Blood From Healthy Volunteers and Patients With Hereditary Angioedema Due to C1 Inhibitor Deficiency
Source: Front Immunol. 2018 Oct 2;9:2183. doi: 10.3389/fimmu.2018.02183 (PMC6176197; doi:10.3389/fimmu.2018.02183)
Supplement: Supplementary file 1 [file Data_Sheet_1.PDF]

Supplementary Material

Comparing pathways of bradykinin formation in whole blood from healthy volunteers and patients with hereditary angioedema due to C1 inhibitor deficiency

Xavier Charest-Morin, Jacques Hébert, Georges-Étienne Rivard, Arnaud Bonnefoy, Eric Wagner, François Marceau

*** Correspondence:** F. Marceau: francois.marceau@crchudequebec.ulaval.ca

Figure S1. Validation of an expression vector for human B_1R using the binding of the cognate ligand [^{3}H]Lys-des-Arg^9-BK in adherent HEK 293a cells that transiently express this receptor in 24-well plates. Values composed of duplicate determinations. Inset: Scatchard plot to determine binding parameters (K_D = 0.50 nM, B_{max} = 27 fmol/well). The human B_1R sequence was cloned from the previously reported B_1R-FLAG vector (25) using the PCR primers 5'-cgtttaaacgggccctATGGCATCATCCTGGCCC-3' (forward) and 5'-ttggtaccgagctcgTTAATTCCGCCAGAAAAGTTGG-3' (reverse). Using the Gibson assembly technique, both fragments were ligated in the XbaI/BamHI digestion product of the pcDNA3.1 vector to generate the human B_1R vector. The insert coding for B_1R was validated by automated sequencing.

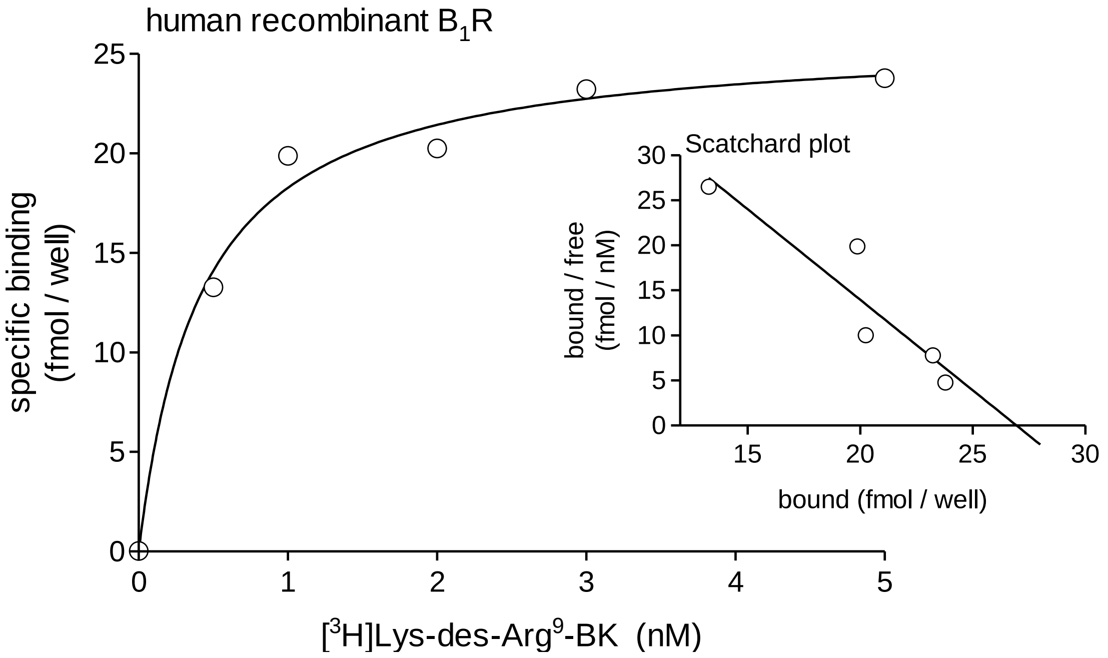

Figure S2. Immunoreactive BK measured in extracts of fresh human citrated blood from healthy donors incubated at 37°C under mild agitation in the presence of IL-8, with optional addition of enalaprilat 130 nM (both applied at time zero). Values are means ± S.D. and are all similar to the controls reported in Fig. 3. Presentation as in Fig. 3.

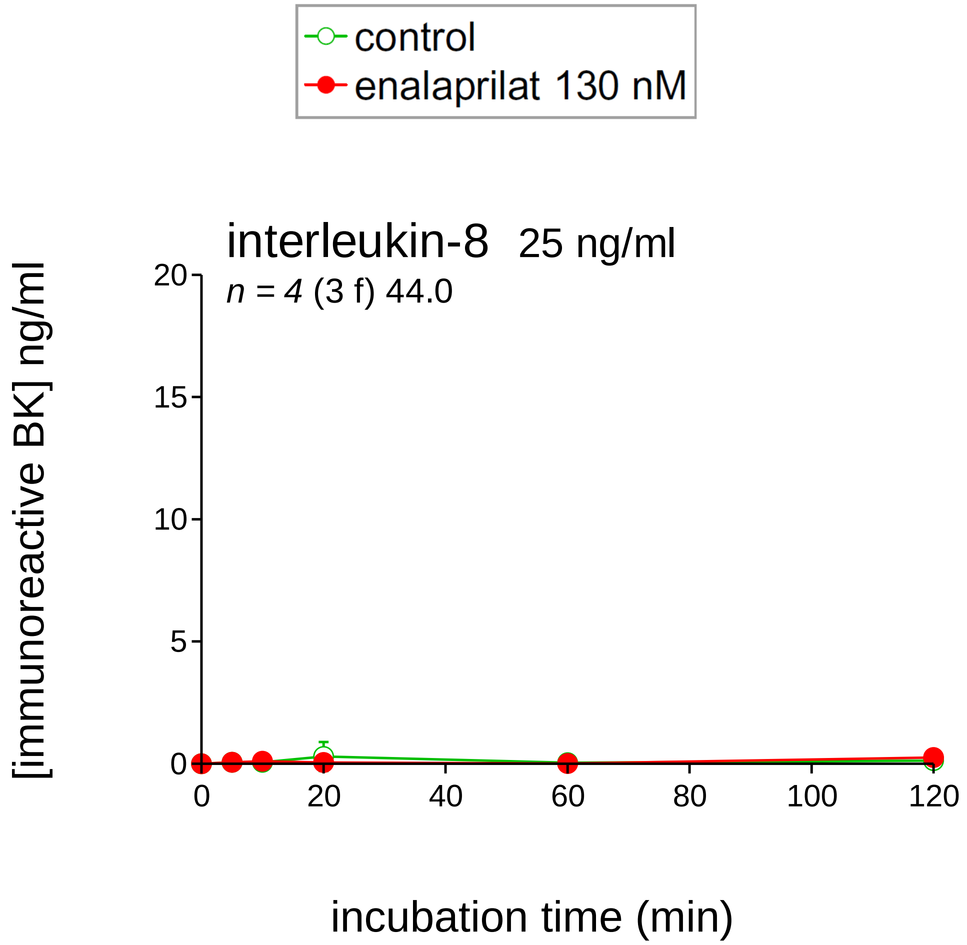

Figure S3. Cross-reactivity of the BK EIA with selected kinin sequences. The inactive metabolite des-Arg^1-BK (= BK fragment 2-9) exhibits full cross-reactivity *vs.* BK whereas the optimal agonist of the B_1R, Lys-des-Arg^9-BK, exhibits none.

-

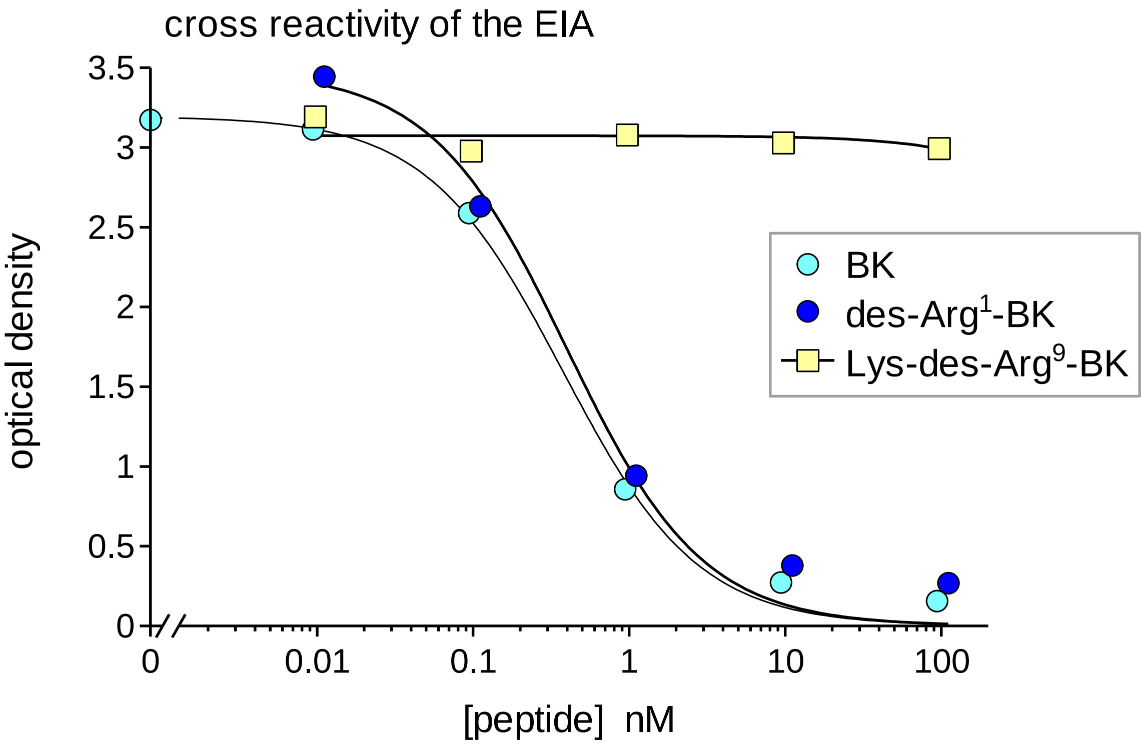

Figure S4. c-Fos accumulation in HEK 293a cells expressing recombinant B_2Rs (A) or B_1Rs (B) and stimulated for 60 min with synthetic peptides, as indicated.

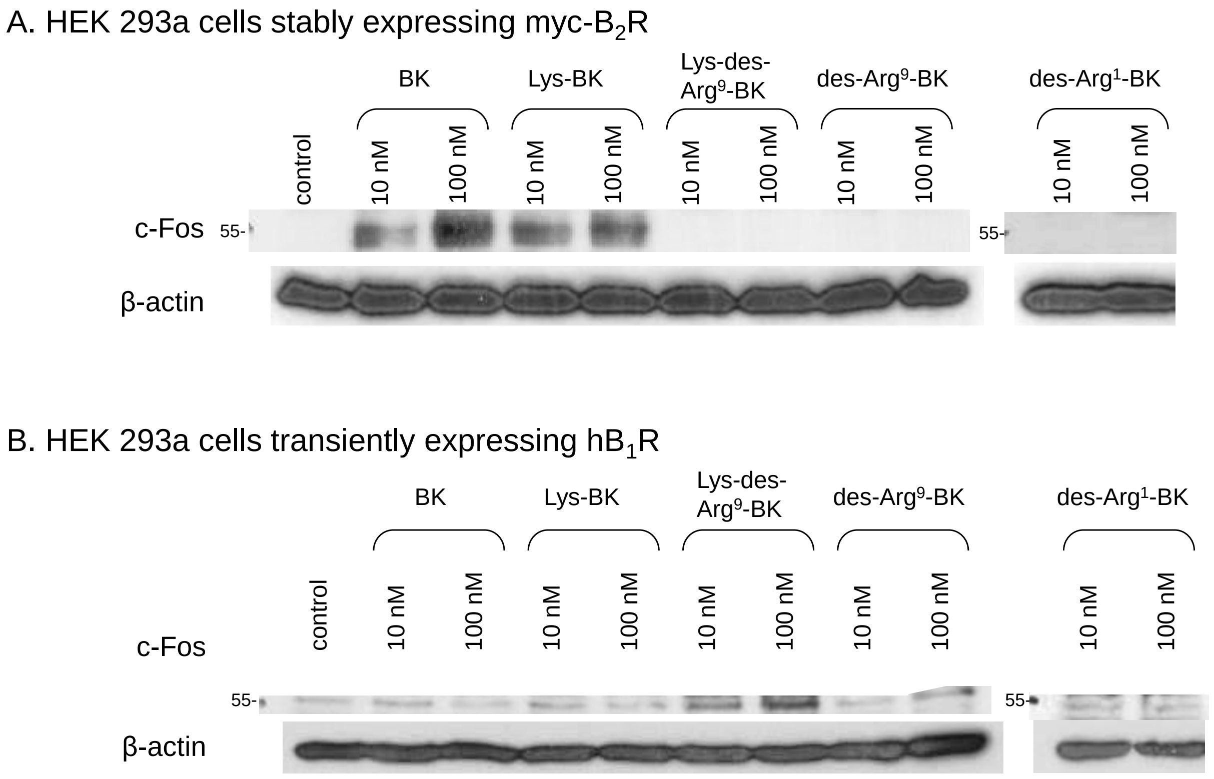

Table S1. Immunoreactive BK concentrations measured in citrated blood samples (total volume 1 ml) submitted to various forms of stimulation in the aggregometer. All stimulations induced platelet aggregation except for kaolin.

Enalaprilat preteatment	treatment	incubation time (min)	[iBK] ng/ml in extract
1 µM	collagen 4 µg/ml	0	0
		5	0
		15	0
	thrombin receptor-activating peptide (TRAP-6) 10 µM	0	0
		5	1.4
		15	0
	collagen 4 µg/ml + TRAP 10 µM	0	0
		5	0
		15	1.6
10 µM	collagen 4 µg/ml	0	0
		5	0
		15	0
1 µM	kaolin 20% v/v (CK-Prest Stago reagent)	0	0.2
		5	56.
		15	20.
